# Bromide of Strontium in Epilepsy

**Published:** 1892-06

**Authors:** 


### Selections and Abstracts.

### BROMIDE OF STRONTIUM IN EPILEPSY.

Interesting tests of this salt in epilepsy were made by Dr. Ch. Fere who reported his results to the Paris Biological Society on October 21st, 1891. M. Fere first administered the medicament to a series of epileptics who had long been treated by the bromide of potassium and whose condition had been ameliorated by the use of the latter salt. But he had found that after the potassium bromide treatment, and in spite of the fact that there had been an amelioration in a general way, the patients were subject to serious relapses and that grave, nervous crises supervened. He found that after using the bromide of strontium, these relapses and crises did not appear, and he clearly states his conviction that this salt should supplant the bromide of potassium in the treatment of epilepsy. Concerning the physiological effects of the two salts, Dr. Fere remarked that he found that the strontium bromide exerted about five times less toxic power than that of the potassium salt. This observer also developed the important fact that a given quantity of the bromide of strontium produced the same therapeutic effect as the same amount of bromide of potassium. This is the more remarkable as the former contains about one third less bromine than the latter, or, in other words, 1 1-2 grains of the strontium salt contains the same amount of bromine as 1 grain of bromide of potassium. Of course, other things being equal, the advantage would lie with the medicament which would give the better results with the smaller quantity of the metalloid. Like other observers, M. Fere insists upon the purity of the medicament, which he considers to be an absolute *sine qua non* for therapeutic efficiency, and, for his own tests, he made exclusive use of the bromide of strontium (Paraf-Javal.)

